# Severe COVID-19 acute respiratory distress syndrome in an adult with single-ventricle physiology: a case report

**DOI:** 10.1186/s12871-021-01504-5

**Published:** 2021-11-13

**Authors:** Götz Schmidt, Christian Koch, Matthias Wolff, Michael Sander

**Affiliations:** Department of Anaesthesiology, Intensive Care Medicine and Pain Therapy, Justus Liebig University of Giessen, Rudolf-Buchheim-Strasse 7, 35392 Giessen, Germany

**Keywords:** Tricuspid valve atresia, Transposition of the great arteries, SARS-CoV-2, Perioperative, Bidirectional cavopulmonary shunt

## Abstract

**Background:**

COVID-19 can induce acute respiratory distress syndrome (ARDS). In patients with congenital heart disease, established treatment strategies are often limited due to their unique cardiovascular anatomy and passive pulmonary perfusion.

**Case presentation:**

We report the first case of an adult with single-ventricle physiology and bidirectional cavopulmonary shunt who suffered from severe COVID-19 ARDS. Treatment strategies were successfully adopted, and pulmonary vascular resistance was reduced, both medically and through prone positioning, leading to a favorable outcome.

**Conclusion:**

ARDS treatment strategies including ventilatory settings, prone positioning therapy and cannulation techniques for extracorporeal oxygenation must be adopted carefully considering the passive venous return in patients with single-ventricle physiology.

## Background

The global spread of the novel severe acute respiratory syndrome coronavirus 2 (SARS-CoV-2) has resulted in the widespread occurrence of coronavirus disease 2019 (COVID-19), which typically presents with respiratory symptoms and fever. However, COVID-19 can induce acute respiratory distress syndrome (ARDS) and organ dysfunction or failure. According to the position paper from the ESC working group of adult congenital heart disease, and the International Society for Adult Congenital Heart Disease, patients with univentricular heart, complex uncorrected or palliated cardiac anatomy, cyanosis and significant valve disease may be at highest risk for adverse outcomes [1]. In patients with congenital heart disease, established treatment strategies are often limited due to their unique cardiovascular anatomy. Up to now, there is only one report of an adult with single-ventricle physiology and moderate COVID-19 not requiring endotracheal intubation [2]. We report the first case – to our knowledge - of severe COVID-19-associated ARDS in an adult with single-ventricle physiology after bidirectional cavopulmonary shunt (BCPS) with additional pulmonary blood flow. Consent for publication was obtained from the patient’s legal representative.

## Case presentation

**Fig. 1:**
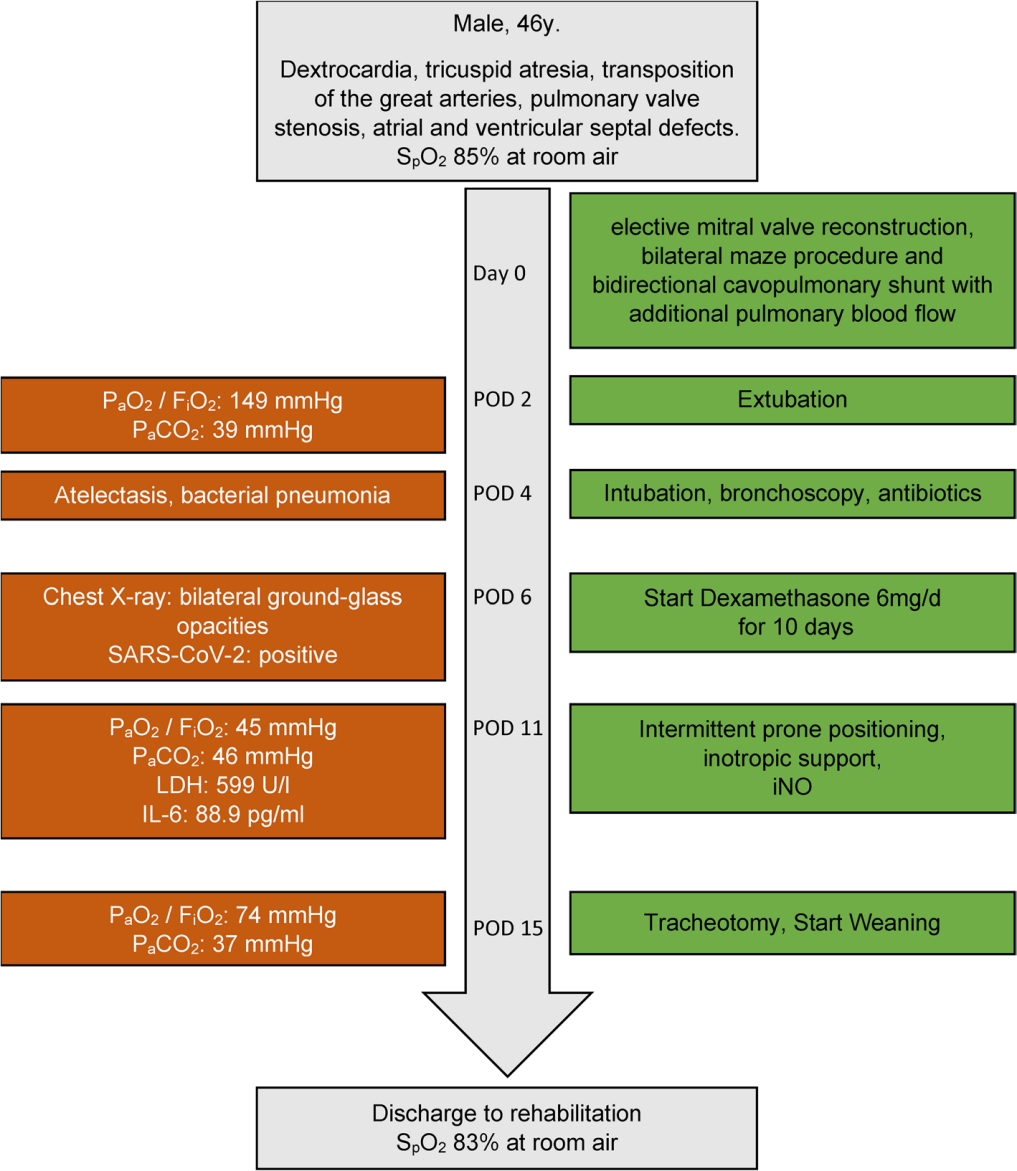
Timeline. (POD = postoperative day, iNO = inhalative nitric oxide)

**Fig. 2:**
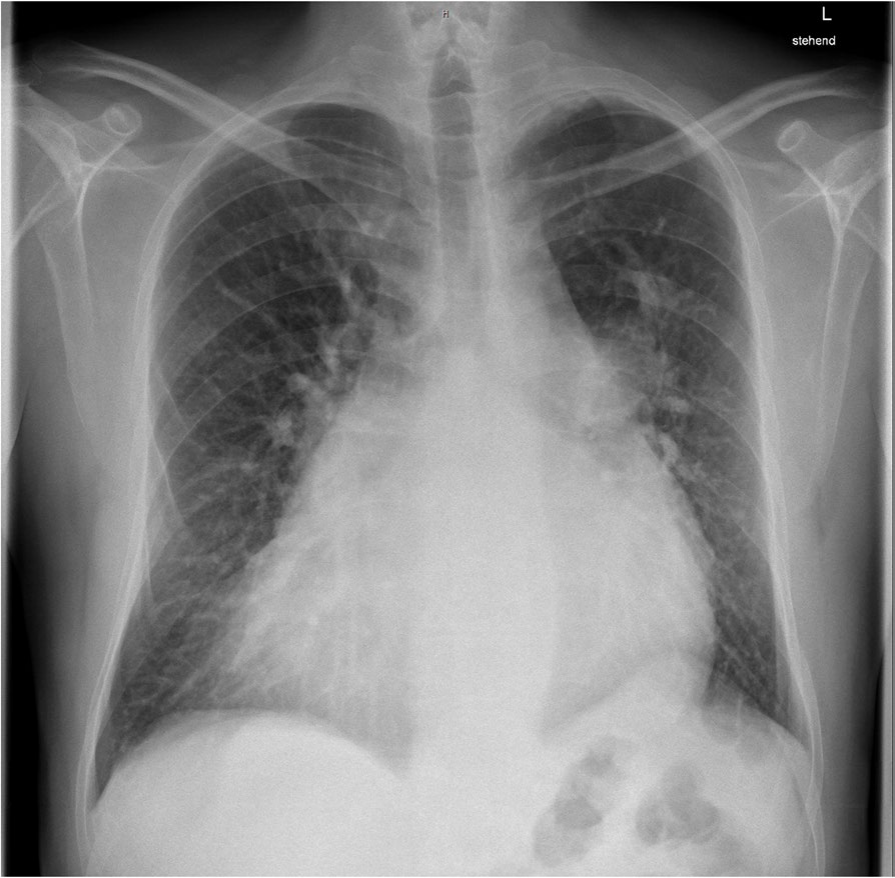
Preoperative chest X-ray of a 46-year-old man with congenital heart disease shows dextrocardia, cardiomegaly and no opacities or pleural effusion

A 46-year-old man with dextrocardia, unpalliated tricuspid atresia, transposition of the great arteries, pulmonary valve stenosis, and atrial and ventricular septal defects presented with shortness of breath, recurrent syncopal attacks, and chronic atrial fibrillation (Fig. 1). At that time, chest X-ray showed no opacities (Fig. 2) and SARS-CoV-2 screening was not recommended for routine clinical practice. Heart catheterization revealed satisfying pulmonary hemodynamic data (mean pulmonary venous wedge pressure 15 mmHg, pulmonary vascular resistance 199.67 dynes*sec/cm^5^). Consequently, due to severe mitral insufficiency and a highly enlarged atrium, the patient underwent elective mitral valve reconstruction, bilateral maze procedure and bidirectional cavopulmonary shunt with additional pulmonary blood flow (also known as pulsatile bidirectional Glenn). The right pulmonary artery (RPA) was narrowed with a fenestrated pericardial patch, but pulsatile blood flow to the left pulmonary artery was preserved. Due to right-to-left shunt, an arterial oxygen saturation (S_a_O_2_) of 83% and a partial pressure of oxygen (p_a_O_2_) of 47 mmHg were considered satisfactory. On postoperative day (POD) 4, reintubation was necessary due to atelectasis and bacterial pneumonia. Atelectasis was opened by bronchoscopy, and pneumonia was treated.

**Fig. 3:**
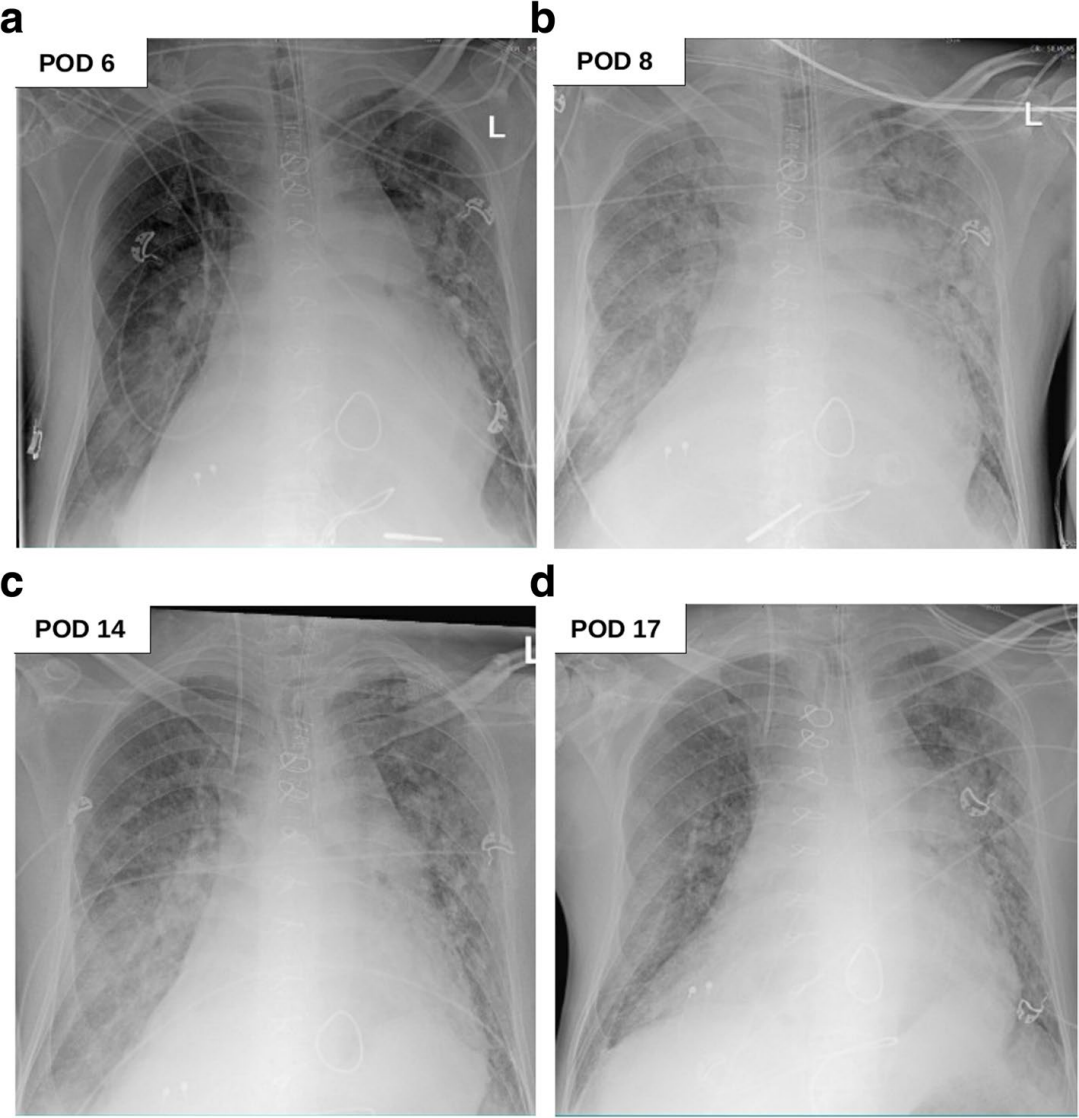
After elective mitral valve reconstruction, bilateral maze procedure and bidirectional cavopulmonary shunt with additional pulmonary blood flow, bilateral ground-glass opacities led to severe COVID-19 ARDS at POD 6. (**a, b**) The patient was treated with intermittent prone positioning and inhalative nitric oxide. This led to a recovery of the patients’ lung (**c, d**). (COVID-19 = coronavirus disease 2019, ARDS = acute respiratory distress syndrome, POD = postoperative day)

Due to suspicious X-ray findings, SARS-CoV-2 reverse transcriptase-polymerase chain reaction assay was performed on bronchoalveolar lavage and nasopharyngeal swab, yielding a positive result on POD 6 (Fig. 3a). Dexamethasone (6 mg/d) was administered for 10 days, and therapeutic anticoagulation was continued with unfractionated heparin. However, oxygenation deteriorated until an S_a_O_2_ of 76% and a p_a_O_2_ of 45 mmHg could only be maintained with pressure-controlled ventilation at a fraction of inspired oxygen of 100% and 10 mbar positive end-expiratory pressure (PEEP). Chest X-ray showed bilateral ground-glass opacities; thus, severe ARDS was diagnosed (Fig. 3). Simultaneously, significant increases in lactate dehydrogenase (599 U/l) and interleukin-6 (188.9 pg/ml) were noted. Heart frequency lower than 100 bpm, mean arterial pressure of 70 mmHg, S_a_O_2_ of 80% and cerebral oxygen saturation above 35% measured with near-infrared spectroscopy were considered as surrogates for sufficient cerebral blood flow and cardiac output. Tidal volume was kept between 500 and 650 ml (6 - 8 ml/kg body weight). Unfortunately, no further increase in PEEP was possible due to increased venous congestion and decreased cardiac output following a reduction of venous return (VR). To counteract these effects, inhaled nitric oxide (NO) therapy was initiated for pulmonary vasodilation. Levosimendan (0.05 μg/kg/min) ensured additional inotropic support. Mean arterial pressure was maintained with noradrenaline (maximum rate of 0.1 μg/kg/min) and vasopressin (maximum rate of 2 I.E./h). Extracorporeal membrane oxygenation (ECMO) was considered as last resort treatment. Intermittent prone positioning was utilized for 16 h each day, which improved the patient’s oxygenation. After further intensive treatment with tracheotomy, weaning proceeded without abnormalities, and the patient was discharged to rehabilitation in good neurological condition. The patient is followed-up regularly at the outpatient clinic for adults with congenital heart disease. Today, he presents in stable general condition and his oxygen saturation is 83% at rest.

## Discussion and conclusions

In patients with BCPS, the superior vena cava (SVC) is directly connected to the RPA. Therefore, pulmonary perfusion strongly depends on VR. The pulmonary and systemic bed are partly connected in series; thus, VR defines the preload and cardiac output (CO) of the ventricle. VR from the SVC is a passive process – secondary driven by the single ventricle’s CO - that is largely hampered by intrathoracic pressure and pulmonary vascular resistance (PVR). Only a small portion of pulmonary perfusion is ensured by antegrade blood flow; thus, the management of our patient was complicated by the fact that in patients with single-ventricle physiology common principles of ventilatory settings are opposite to those with ARDS. Patients with passive pulmonary perfusion benefit from lower PEEP, lower mean airway pressures with lower respiratory rates and slightly higher tidal volumes. In contrast, low tidal volumes (6 ml/kg ideal body weight) and high respiratory rates are regularly used to treat ARDS. Furthermore, the application of higher PEEP up to 24 cmH_2_O is particularly important in typical ARDS [3, 4]. PEEP improves alveolar recruitment and reduces shunt but also enhances intrathoracic pressure and PVR [5]. Compared with spontaneously breathing patients, the cardiac index was found to be reduced in ventilated patients with BCPS, associated with lower cerebral oxygen saturation, which suggested that decreased VR may provoke cerebral congestion and damage [6]. Similar considerations apply to the hemodynamic effects of prone positioning. However, the recruitment of dorsal lung areas improves ventilation/perfusion mismatch; therefore, prone positioning improves oxygenation and reduces mortality [5, 7]. However, in our case, atelectasis recruitment through prone positioning and ventilation with moderate PEEP reduced PVR, leading to an improved VR and CO. This may be explained by the fact, that PVR is increased by atelectasis, hypoxia and acidosis following hypercapnia. Thus, we supposed that our treatment strategy lowered PVR in total to a larger extent rather than it would be increased by PEEP and prone positioning by itself. Therefore, RPA pressure was carefully monitored through the right internal jugular vein. However, evidence has recently emerged, that severe hypoxemia in COVID-19 ARDS is driven by significant ventilation-perfusion mismatch, to larger extent rather than by atelectasis in typical ARDS. Several studies observed pathological vasodilatation and diffuse alveolar damage leading to extensive hyperperfusion of poorly ventilated areas [8, 9]. Autopsy studies revealed prominent intrapulmonary bronchopulmonary anastomoses (IBA) generating shunt unrelated to atelectasis that cannot be recruited by PEEP [10]. In this context, hence applying higher PEEP could do more harm than good through overdistension of less affected lung regions hampering its mechanics and triggering further damage and fibrosis. Obviously, in patients with preexisting right-to-left shunt, additional shunt volume through IBA and severe ventilation-perfusion inequality may lead to faster deterioration of oxygen delivery with consecutive organ failure that is only hardly improved by established treatment strategies. In our patient, cerebral oxygen saturation was also measured to detect relevant cerebral congestion, which may have been prevented by the fenestrated pericardial patch offering additional outflow to the left pulmonary artery if intrathoracic pressure exceeded. Moreover, obtaining antegrade blood flow from the single ventricle into the left pulmonary artery made the patient’s circulation not solely dependent on passive venous flow which may helped obtaining cardiac output. Intermittent prone positioning improved the patient’s oxygenation; thus, ECMO therapy was not mandatory. Otherwise – in case of hemodynamic failure due to impaired VR during prone positioning and positive pressure ventilation - it would have been initiated. In patients with BCPS, venovenous ECMO therapy may be realized via femoral cannulation using single lumen cannulas draining blood from inferior vena cava and reinfusing in common atrium. Recently, double lumen cannulas combining drainage and reinfusion in one cannula have been introduced. However, both strategies are sufficient only to normal values of PVR. Otherwise, venous congestion must be prevented using additional venous drainage from the jugular vein to drain blood from the separated SVC [11]. Furthermore, this specific patient population may benefit from early initiation of awake ECMO therapy, thus avoiding intubation and positive-pressure ventilation. Avoiding the above-mentioned effects of moderate PEEP, physiological negative intrathoracic pressures supporting VR were obtained. It must be noticed, that – as in our case - therapeutic decisions should be made after interdisciplinary team discussions involving anesthesiologists, intensive care physicians, pediatric cardiologists, cardiac surgeons and perfusionists.

Fluid overload is a common reason for hypoxemia in cardiac patients, and not only in the postoperative phase. Likewise, lung ultrasound could be helpful in such patients to determine the cause of hypoxemia not only showing B-lines as unspecific sign of interstitial syndrome as well as revealing specific features of irregular pleural lines in COVID-19 pneumonia [12, 13]. Furthermore, a high incidence of venous thrombosis and undetected pulmonary embolism has been reported in COVID-19 autopsy findings, underlining the necessity of an effective anticoagulation strategy, as even pulmonary microembolism can increase PVR [14]. Moreover, due to the passive venous drainage from the SVC, patients with BCPS are constantly at risk for venous thrombosis, which might be exacerbated by COVID-19-induced coagulopathy. Summarizing, we successfully adopted established ARDS treatment strategies and reduced PVR, both medically and through prone positioning. However, ventilatory settings, prone positioning therapy and cannulation techniques for extracorporeal oxygenation must be considered carefully regarding the passive venous return. Further research investigating the values of specific strategies for the treatment of COVID-19 ARDS in patients with single-ventricle physiology may be warranted.

## Data Availability

The datasets used and/or analysed during the current study are available from the corresponding author on reasonable request.

## Abbreviations

ARDS: Acute respiratory distress syndrome
BCPS: Bidirectional cavopulmonary shunt
CO: Cardiac output
COVID-19: Coronavirus disease 2019
ECMO: Extracorporeal membrane oxygenation
IBA: Intrapulmonary bronchopulmonary anastomoses
NO: Nitric oxide
paO2: Arterial partial pressure of oxygen
PEEP: Positive end-expiratory pressure
POD: Postoperative day
PVR: Pulmonary vascular resistance
RPA: Right pulmonary artery
SVC: Superior vena cava
SaO2: Arterial oxygen saturation
SARS-CoV-2: Severe acute respiratory syndrome coronavirus 2
VR: Venous return
